# Patterns in Geographic Distribution of Substance Use Disorder Treatment Facilities in the US and Accepted Forms of Payment From 2010 to 2021

**DOI:** 10.1001/jamanetworkopen.2022.41128

**Published:** 2022-11-11

**Authors:** Jonathan H. Cantor, Maria DeYoreo, Russell Hanson, Aaron Kofner, David Kravitz, Adrian Salas, Bradley D. Stein, Kandice A. Kapinos

**Affiliations:** 1 RAND Corporation, Santa Monica, California; 2 RAND Corporation, Arlington, Virginia; 3 RAND Corporation, Pittsburgh, Pennsylvania; 4 Peter J. O’Donnell School of Public Health, University of Texas Southwestern Medical Center, Dallas

## Abstract

**Importance:**

The drug overdose crisis is a continuing public health problem and is expected to grow substantially in older adults. Understanding the geographic accessibility to a substance use disorder (SUD) treatment facility that accepts Medicare can inform efforts to address this crisis in older adults.

**Objective:**

To assess whether geographic accessibility of services was limited for older adults despite the increasing need for SUD and opioid use disorder treatments in this population.

**Design, Setting, and Participants:**

This longitudinal cross-sectional study obtained data on all licensed SUD treatment facilities for all US counties and Census tracts listed in the National Directory of Drug and Alcohol Abuse Treatment Programs from 2010 to 2021.

**Main Outcomes and Measures:**

Measures included the national proportion of treatment facilities accepting Medicare, Medicaid, private insurance, or cash as a form of payment; the proportion of counties with a treatment facility accepting each form of payment; and the proportion of the national population with Medicare, Medicaid, private insurance, or cash payment residing within a 15-, 30-, or 60-minute driving time from an SUD treatment facility accepting their form of payment in 2021.

**Results:**

A total of 11 709 SUD treatment facilities operated across the US per year between 2010 and 2021 (140 507 facility-year observations). Cash was the most commonly accepted form of payment (increasing slightly from 91.0% in 2010 to 91.6% by 2021), followed by private insurance (increasing from 63.5% to 75.3%), Medicaid (increasing from 54.0% to 71.8%), and Medicare (increasing from 32.1% to 41.9%). The proportion of counties with a treatment facility that accepted Medicare as a form of payment also increased over the same study period from 41.2% to 53.8%, whereas the proportion of counties with a facility that accepted Medicaid as a form of payment increased from 53.5% to 67.1%. The proportion of Medicare beneficiaries with a treatment facility that accepted Medicare as a form of payment within a 15-minute driving time increased from 53.3% to 57.0%. The proportion of individuals with a treatment facility within a 15-minute driving time that accepted their respective form of payment was 73.2% for those with Medicaid, 69.8% for those with private insurance, and 71.4% for those with cash payment in 2021.

**Conclusions and Relevance:**

Results of this study suggest that Medicare beneficiaries have less geographic accessibility to SUD treatment facilities given that acceptance of Medicare is low compared with other forms of payment. Policy makers need to consider increasing reimbursement rates and using additional incentives to encourage the acceptance of Medicare.

## Key Points

**Question:**

What proportion of the national population has local access to a licensed substance use disorder (SUD) treatment facility that accepts Medicare, Medicaid, and private insurance or cash as a form of payment?

**Findings:**

In this cross-sectional study of 11 709 SUD treatment facilities (140 507 facility-year observations) in the US between 2010 and 2021, the proportion of Medicare beneficiaries with a treatment facility that accepted Medicare as a form of payment and was within a 15-minute driving time increased. In contrast, individuals with Medicaid, private insurance, and cash payment had greater geographic access to a treatment facility that accepted their form of payment in both 2010 and 2021.

**Meaning:**

Findings of this study suggest that Medicare beneficiaries have less access to SUD treatment facilities given the low acceptance of Medicare compared with other forms of payment.

## Teaser

This cross-sectional study examines the use of Medicare as a form of payment among drug and alcohol use treatment programs across the US.

## Introduction

There has been a drastic increase in the number of drug overdose deaths in the US since 1999.^1^ However, such increases are not uniformly distributed across the country. There are large differences in the relative increase in drug overdose deaths across counties between 1980 and 2014, with geographic disparities widening over time.^2^ Previous evidence has found an association between geographic disparities and differences in local unemployment rate, poverty rate, educational levels, and urbanicity.^3^ A separate but related concerning pattern is the increase in opioid-related deaths among older adults. In the US, 45% of all opioid-related deaths involve individuals 45 years or older, and since 1999, drug overdose death rates have increased the most (by 6-fold) among those aged 55 to 64 years compared with other age groups^4^ despite opioid misuse being lower among older adults than younger adults.^5^ High overdose rates may be due to prescription opioids being more likely to be prescribed to adults 50 years or older than to younger adults^6,7^ or due to the risk of overdose being greater with age because of changes in metabolism and the prevalence of chronic disease.^8^

The number of older adults receiving treatment for opioid use disorder (OUD) has almost doubled since 2007.^9^ Yet, in 2018, the proportion of Medicare beneficiaries who received treatment for an identified substance use disorder (SUD) was only 11%.^10^ Two of the most commonly cited reasons for not receiving care were related to access: limited affordability and limited availability of treatment.^10,11^ For both of these aspects, there are unique challenges to treating SUD and OUD among older adults. One barrier is that not all SUD treatment facilities accept Medicare, the most common insurance coverage for those 65 years or older, as a form of payment. In 2017, only 35% of treatment facilities accepted Medicare.^12^ Possible reasons included Medicare lacking a reimbursement rate for intensive outpatient services and not allowing SUD treatment to be provided as part of the home health care benefit.^10^ Another barrier is that older adults may face greater access challenges given that approximately 25% of adults older than 65 years live in a small town or rural area; this rate is higher than for younger adults.^13^ In general, rural areas tend to have fewer SUD treatment facilities,^14^ and rural county residents have less use of medications for OUD.^15^ Previous evidence has suggested an association between fewer SUD treatment resources in rural regions and limited economic growth, lower median income, and higher rates of poverty than their urban counterparts.^16^

Even though the number of Medicare beneficiaries with SUD is expected to grow rapidly,^17^ relatively little is known about the geographic availability of SUD treatment. To better understand the evolution of geographic accessibility of SUD treatment facilities, we used a national panel data set of SUD treatment facilities between 1975 and 2021 to quantify changes in the acceptance of Medicare, Medicaid, private insurance, and cash payment as forms of payment from 2010 to 2021. We compared geographic access to facilities accepting Medicare vs other forms of payment to assess whether geographic accessibility of services was limited for older adults despite the increasing need for SUD and OUD treatments in this population.

## Methods

The RAND Institutional Review Board deemed this study exempt from review and the requirement for informed consent because it was considered to be nonhuman participant research. We followed the Strengthening the Reporting of Observational Studies in Epidemiology (STROBE) reporting guideline.

### Data Sources

This cross-sectional study used the Mental Health and Addiction Treatment Tracking Repository (MATTR) data set, which was obtained from various annual versions of the National Directory of Drug and Alcohol Abuse Treatment Programs. The directories contain the treatment facilities that voluntarily responded to the National Survey of Substance Abuse Treatment Services (N-SSATS) and agreed to be listed in the directory between 1975 and 2021. We used the directories with N-SSATS data from 2010 to 2021.

Each directory listing included facility-level detail, such as whether the facility was licensed, certified, or otherwise approved for inclusion in a national directory by a state substance abuse service agency that responded to the previous year’s N-SSATS. The data excluded unlicensed facilities or facilities that chose not to be listed in the directories. The directories for most years included information on the forms of payment accepted by the facility and the street address of the facility. As has been done in other studies,^18^ we linked the facilities across years according to their address using geocoding (ArcMap, version 10.8; Esri) and creating a longitudinal panel of facilities over time. Between 1975 and 2021, 96% of treatment facility listings were geocoded at the address level and 4% were geocoded at the zip code level.

We linked the MATTR facility-level data to the American Community Survey 5-year estimates at the Census tract level.^19^ The American Community Survey data included the proportion of people in a Census tract who had Medicare coverage only, Medicaid or means-tested public coverage only, employer-based health insurance only, direct-purchase coverage only, and no health insurance coverage.

### Measures

We created several measures of access for each year from 2010 to 2021 using the linked data aggregated at 3 levels: facility, county, and populations by insurance coverage. First, using the sample of all SUD treatment facilities, we calculated the annual national proportion of treatment facilities that accepted Medicare, Medicaid, private insurance, and cash as a form of payment from 2010 to 2021. Second, using the county-level data, we calculated the annual national proportion of counties with at least 1 facility that accepted each form of payment. Simply examining whether there is a treatment facility in the county can mask important aspects of access. For example, there may not be a facility in a county, but there could be one in an adjacent county that is within a 10-minute driving time. Thus, we created another set of measures following the procedure of an existing study.^20^ Third, using the facility-level data, we calculated the percentage of the population in each county who were covered by different insurance types (Medicare, Medicaid, or private) and resided within a 15-minute, 30-minute, or 60-minute driving time from a treatment facility that accepted their insurance or cash payment in 2021. ArcMap 10.8 Network Analyst was used to create network travel time and distance measures between SUD treatment facilities and the centroid of each Census block group in the US.

### Statistical Analysis

Descriptive statistics for each of the measures were calculated for each year. The MATTR data set was created between April 2018 and April 2021. All analyses were conducted between November 2020 and April 2021.

To ascertain whether there were significant changes over time in the proportion of facilities accepting each form of payment, we used linear regression models for the probability of accepting each form of payment, with year as a covariate. We also tested whether the proportion of facilities accepting each form of payment in 2010 was equal to the proportion in 2021. We calculated 95% CIs for the estimated change in proportions between 2010 and 2021. Two-sided tests and *P* < .05 were used to indicate statistical significance.

## Results

The analysis included 140 507 facility-year observations in the MATTR database. There were 11 709 SUD treatment facilities across the US per year (2010-2021) on average.

**Figure 1.**
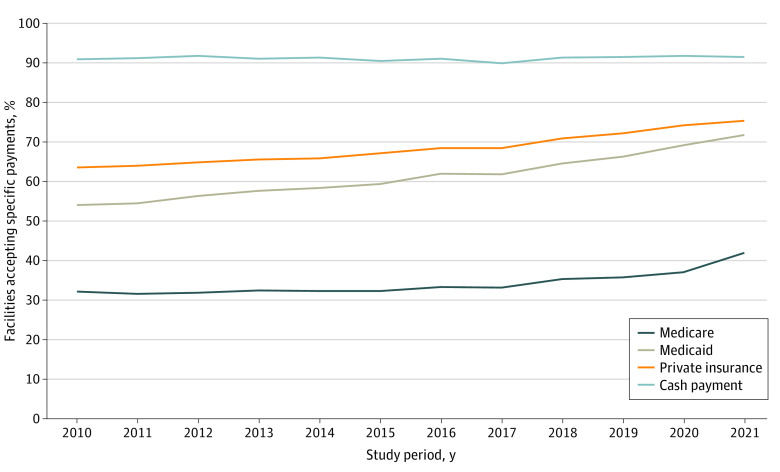
Percentage of Substance Use Disorder Treatment Facilities Accepting Medicare, Medicaid, Private Insurance, and Cash Payment Between 2010 and 2021

Among all facilities in 2010, the most common forms of payment accepted by treatment facilities were cash payment (91.0%), private insurance (63.5%), and Medicaid (54.0%), and the least common form of payment accepted was Medicare (32.1%). By 2021, cash remained the most commonly accepted form of payment, with 91.6% of facilities accepting cash from patients. The proportion of facilities accepting each form increased to 75.3% for private insurance, 71.8% for Medicaid, and 41.9% for Medicare. Despite an increase in the number of facilities accepting these types of payment, those accepting Medicare still lagged. These national patterns are shown in Figure 1. These changes were statistically significant for all insurance types except cash payment, with *P* < .001 when testing whether the proportions in 2010 equaled the proportions in 2021 and *P* < .001 for the regression coefficient for the year in a linear regression. In addition, the point estimates and 95% CIs for the change between 2010 and 2021 were 0.5% (95% CI, −1.8% to 1.25%) for cash payment, 17.8% (95% CI, 16.6%-19.0%) for Medicaid, 9.8% (95% CI, 8.6%-11.1%) for Medicare, and 11.8% (95% CI, 10.7%-13.0%) for private insurance, indicating the largest growth in the proportion of facilities accepting Medicaid.

**Figure 2.**
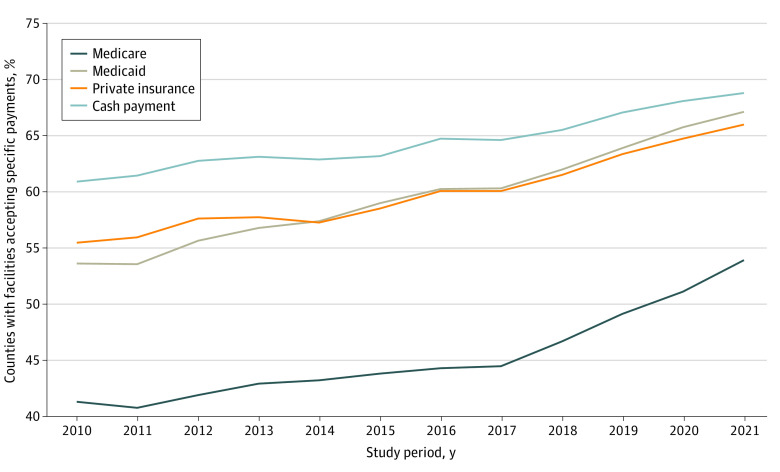
Percentage of Counties With a Substance Use Disorder Treatment Facility Accepting Medicare, Medicaid, Private Insurance, and Cash Payment Between 2010 and 2021

Figure 2 shows the patterns in the proportion of counties with at least 1 treatment facility that accepted the various forms of payment. In 2010, approximately 60.9% of counties had a treatment facility that accepted cash payment, and more than 50% of counties had a facility that accepted either Medicaid (53.5%) or private insurance (55.4%). In contrast, only 41.2% of counties had a treatment facility that accepted Medicare. By 2021, more than 65% of counties had a treatment facility that accepted Medicaid (67.1%), private insurance (65.9%), or cash payment (68.7%), and 53.8% of counties had a facility that accepted Medicare.

**Figure 3.**
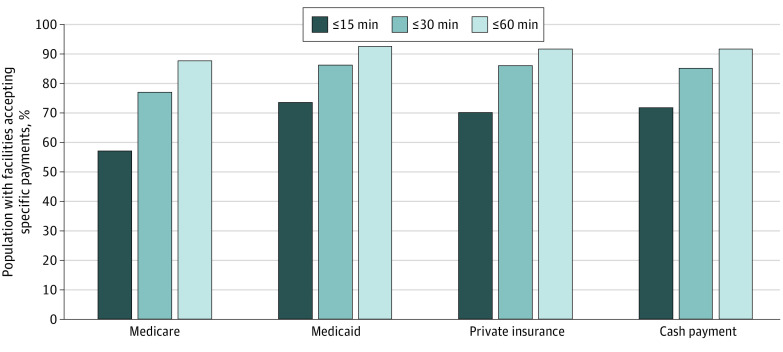
Percentage of the Population With a Substance Use Disorder Treatment Facility Accepting Medicare, Medicaid, Private Insurance, and Cash Payment Within a 15-, 30-, or 60-Minute Driving Time in 2021

The proportion of Medicare beneficiaries with a treatment facility that accepted Medicare as a form of payment and was within a 15-minute driving time increased from 53.3% to 57.0% between 2010 and 2021. In contrast, individuals with Medicaid (69.5% to 73.2%), private insurance (67.0% to 69.8%), and cash payment (71.6% to 71.4%) had greater access to a facility that accepted their form of payment in both years. Figure 3 shows the proportion of the population with Medicare, Medicaid, private insurance, and cash payment with a treatment facility that accepted their form of payment and was within a 15-, 30-, or 60-minute driving time in 2021. Approximately 57.0% of Medicare beneficiaries lived within a 15-minute driving time of a facility accepting Medicare. In contrast, more than 73% of individuals with Medicaid (73.2%), private insurance (69.8%), and cash payment (71.4%) had a facility that accepted their insurance and was within a 15-minute driving time. Nearly all individuals with cash payment (91.3%), private insurance (91.3%), Medicaid (92.2%), and Medicare (87.4%) had a facility within a 60-minute driving time.

**Figure 4.**
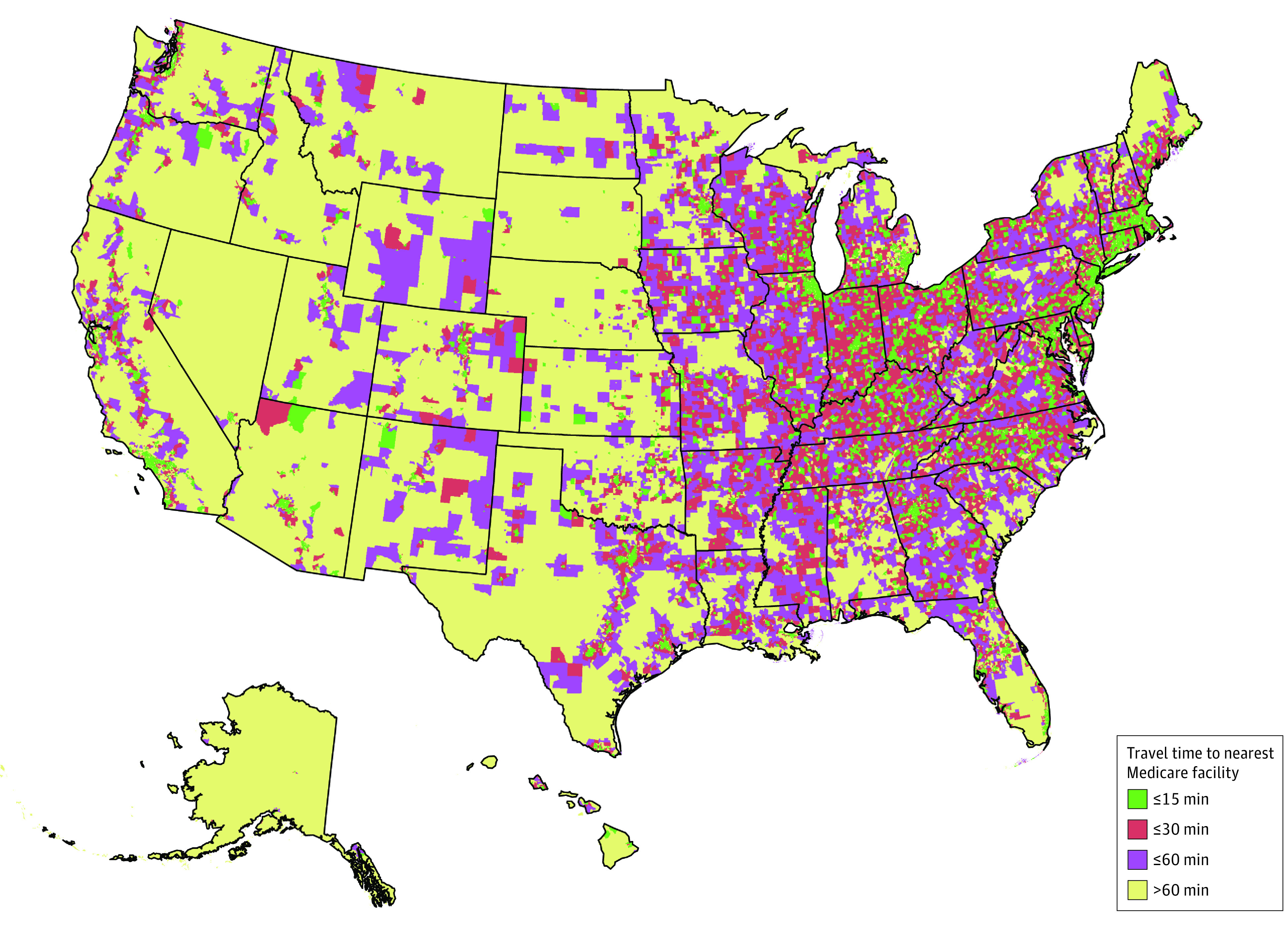
Census Tracts With a Substance Use Disorder Treatment Facility Accepting Medicare Within a 15-, 30-, or 60-Minute Driving Time From the Centroid in 2021 Tracts in yellow lacked a facility within a 60-minute driving time. Tracts in purple contained a facility within a 60-minute driving time. Tracts in green (or red) contained a facility within a 15- (or 30-) minute driving time.

Figure 4 shows the geographic variability in access to a treatment facility that accepted Medicare as a form of payment. Areas that lacked a treatment facility within a 60-minute driving time were concentrated in the Western region, which was less densely populated. In contrast, many of the Census tracts with a facility that accepted Medicare as a form of payment within a 15-minute driving time were on the East Coast, particularly in the more densely populated Northeast region. The eAppendix in the Supplement provides the same map for facilities that accepted Medicaid as a form of payment.

## Discussion

The increase in the number of Medicare beneficiaries with SUD is an urgent public health problem.^10,17^ However, to date, relatively few studies have examined the treatment of SUD and the barriers to receiving treatment for Medicare beneficiaries. In this longitudinal cross-sectional study of licensed SUD treatment facilities in the US between 2010 and 2021, we found that the proportion of facilities accepting different forms of payment (Medicaid, Medicare, private insurance, and cash) increased between 2010 and 2021. However, the percentage of facilities accepting Medicare still lagged behind other payment types, with approximately 42% of facilities accepting Medicare compared with more than 70% accepting Medicaid and private insurance. The smaller number of facilities accepting Medicare as a form of payment may be a factor in the low levels of treatment in this population: only approximately 11% of Medicare beneficiaries with an SUD received treatment.^10^

We also found that, by 2021, a little more than 50% of US counties had a treatment facility that accepted Medicare as a form of payment, whereas more than 65% of counties had facilities that accepted Medicaid and private insurance as a form of payment. Despite increases in the percentage of counties with access to facilities accepting various forms of payments, there remained almost a 15–percentage-point gap between the percentage of counties with at least 1 facility accepting Medicaid and those accepting Medicare. One possible reason for the difference may be that Medicaid provides more comprehensive SUD treatment coverage compared with Medicare. For example, Medicaid covers intensive outpatient or residential treatment for SUD but Medicare does not.^10^

In addition, only approximately 57% of Medicare beneficiaries were within a 15-minute driving time of a facility vs approximately 73% of Medicaid enrollees and individuals with private insurance. Nonetheless, with nearly 25% of Medicare beneficiaries never or rarely driving and 32% of those who drive avoiding driving alone or on highways,^21^ the distance to a facility can be a critical consideration for this population.

The results of this study imply that Medicare is behind other payers in convincing treatment facilities to accept it as a form of payment. Possible reasons include the restrictive rules related to reimbursement or the types of treatment allowed by Medicare. For example, methadone has only recently started being reimbursed by Medicare, which may be a reason that many facilities have not accepted Medicare as a form of payment. Alternatively, the growth in Medicaid acceptance by facilities may be associated with state Medicaid expansions, which have played a role in the increased number and proportion of SUD and OUD treatment facilities that accept Medicaid as a form of payment.^22,23,24^ Although the proportion of facilities that accepted Medicare as a form of payment was lower than the proportion of facilities that accepted other types of insurance, Medicare beneficiaries appeared to have almost an equal amount of geographic access to a facility within a 60-minute driving time as individuals with other forms of insurance.

This study suggests that, between 2010 and 2021, geographic access increased for SUD treatment for all payers. However, the proportion of facilities that accepted Medicare remained low. This finding is particularly concerning given that the number of older adults with SUD has increased drastically.^9^ In addition, SUD treatment for older adults may need to be adjusted to respond to the other needs of older adults. For example, there is a dearth of evidence regarding the implications of buprenorphine and naltrexone for older adults.^9^ Federal officials and policy makers have responded to the lack of SUD treatment access for Medicare beneficiaries. Recently, the Centers for Medicare & Medicaid Services added SUD screening to the Medicare Annual Wellness Visit to identify people who may need additional care.^25^ Similarly, under the Substance Use-Disorder Prevention that Promotes Opioid Recovery and Treatment for Patients and Communities Act of 2018, access to OUD treatment for Medicare beneficiaries is expected to increase by creating the Medicare Part B benefit for OUD treatment programs.^26^ Furthermore, due to the COVID-19 pandemic, several changes were made to Medicare to encourage the use of telehealth in the treatment of OUD.^27^ These changes in policy may be factors in reducing the geographic disparities in SUD treatment. However, to keep up with the sustained increase in the number of older adults with SUD, policy makers need to evaluate these new policy changes and continue to encourage SUD treatment facilities to accept Medicare as a form of payment.

### Limitations

This study has several limitations. First, the directory data included only licensed SUD treatment facilities, excluding approximately 10% of treatment facilities that were unlicensed.^28^ Similarly, clinicians with a buprenorphine waiver were also not included in the directories. Many clinicians with a buprenorphine waiver were located in the same county as an SUD treatment facility.^29^ Because we did not include unlicensed facilities and clinicians with a buprenorphine waiver, the results could be interpreted as either an overestimation or underestimation of access to treatment by insurer. Second, we had no information about the quality of care provided by the treatment facilities. Third, we did not have measures of the number of beds or patients served at each facility.

Fourth, we did not analyze barriers to access. Although individuals may have a facility nearby, they may not be able to access it if there are long waits or other barriers to care. For example, transportation may pose a challenge for individuals without a vehicle. In addition, we were not able to differentiate the travel time to the nearest facility between driving a car and using public transit. Thus, the results may not accurately reflect the travel time in Census tracts in which access to a car was low.^30^ Fifth, we did not examine the types of treatment provided by the facilities, such as medications for OUD and the form of payment accepted by these facilities. Future work should examine the outcomes of these factors. Sixth, we did not examine the differences in geographic availability based on whether the individual has multiple forms of insurance, such as dual eligibility (Medicaid and Medicare or Medicare Advantage special needs plan). Instead, we focused on the primary form of insurance. Thus, the results may be an underestimation of the geographic availability for some populations.

Seventh, the measure of geographic availability we used may be an overestimate. Not all practitioners will necessarily be in individuals’ provider network, and these practitioners may not accept their form of insurance. Eighth, we used the American Community Survey data, which reflect the US population as a whole. We expected that the geographic distribution of individuals with SUD likely differed from the general population.^31^ Because of these limitations, the results can be understood as either an overestimation or underestimation of the geographic availability of SUD treatment.

## Conclusions

The results of this cross-sectional study showed that SUD treatment facilities in certain Census block groups in the US less often accepted Medicare compared with other forms of payment, including Medicaid, private insurance, and cash payment. Reimbursement rates and existing policies related to reimbursement are likely reasons for the limited access to treatment facilities for these individuals. Given that older adults are among the fastest growing groups with SUD, access to treatment for this population is an urgent public policy issue. Policy makers should consider increasing reimbursement rates and using additional incentives to encourage the acceptance of Medicare by SUD treatment facilities.

## Notes

Supplement. **eAppendix.** Census Tracts With a SUD Treatment Facility That Accepts Medicaid as a Form of Payment Within 15-, 30-, 60-Minute Drive Time From Their Centroid in 2021
