# Supplementary material for: Patterns in Geographic Distribution of Substance Use Disorder Treatment Facilities in the US and Accepted Forms of Payment From 2010 to 2021
Source: JAMA Netw Open. 2022 Nov 11;5(11):e2241128. doi: 10.1001/jamanetworkopen.2022.41128 (PMC9652758; doi:10.1001/jamanetworkopen.2022.41128)
Supplement: Supplement. — eAppendix. Census Tracts With a SUD Treatment Facility That Accepts Medicaid as a Form of Payment Within 15-, 30-, 60-Minute Drive Time From Their Centroid in 2021 [file jamanetwopen-e2241128-s001.pdf]

# Supplemental Online Content

Cantor JH, DeYoreo M, Hanson R, et al. Patterns in geographic distribution of substance use disorder treatment facilities in the US and accepted forms of payment from 2010 to 2021. *JAMA Netw Open*. 2022;5(11):e2241128.
doi:10.1001/jamanetworkopen.2022.41128

**eAppendix.** Census Tracts With a SUD Treatment Facility That Accepts Medicaid as a Form of Payment Within 15-, 30-, 60-Minute Drive Time From Their Centroid in 2021

This supplemental material has been provided by the authors to give readers additional information about their work.

© 2022 Cantor JH et al. *JAMA Network Open.*

**eAppendix.** Census Tracts With a SUD Treatment Facility That Accepts Medicaid as a Form of Payment Within 15-, 30-, 60-Minute Drive Time From Their Centroid in 2021

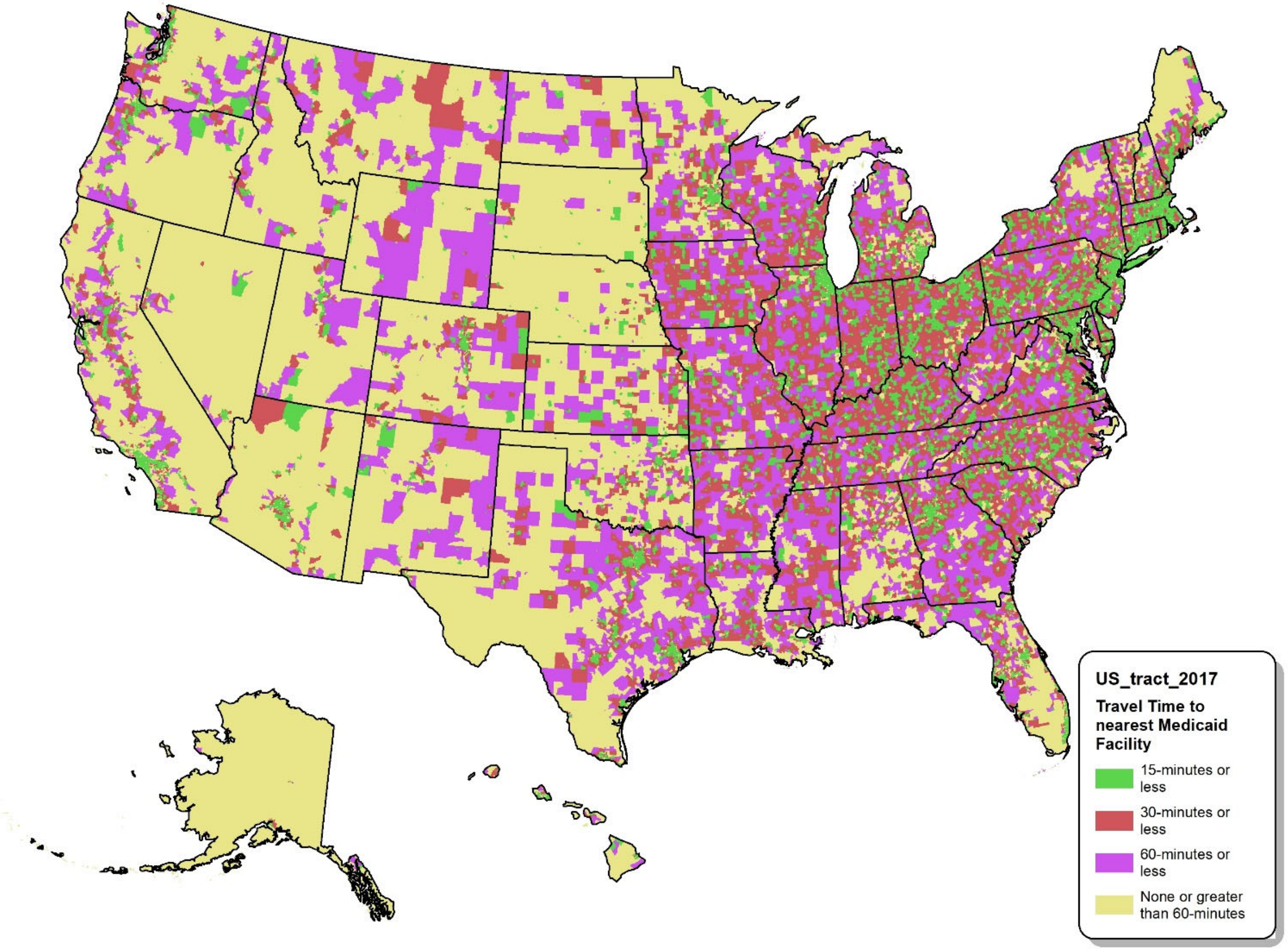


**Notes:** Tracts in yellow lack a treatment facility within a 60-minute drive time. Tracts in purple contain a treatment facility within 60-minutes. Finally, tracts in green (red) contain a treatment facility within a 15-(30-) minute drive time.

© 2022 Cantor JH et al. *JAMA Network Open.*
